# Acute Effects of TiO_2_ Nanomaterials on the Viability and Taxonomic Composition of Aquatic Bacterial Communities Assessed via High-Throughput Screening and Next Generation Sequencing

**DOI:** 10.1371/journal.pone.0106280

**Published:** 2014-08-27

**Authors:** Chu Thi Thanh Binh, Tiezheng Tong, Jean-François Gaillard, Kimberly A. Gray, John J. Kelly

**Affiliations:** 1 Department of Biology, Loyola University Chicago, Chicago, Illinois, United States of America; 2 Department of Civil and Environmental Engineering, Northwestern University, Evanston, Illinois, United States of America; University of Aveiro, Portugal

## Abstract

The nanotechnology industry is growing rapidly, leading to concerns about the potential ecological consequences of the release of engineered nanomaterials (ENMs) to the environment. One challenge of assessing the ecological risks of ENMs is the incredible diversity of ENMs currently available and the rapid pace at which new ENMs are being developed. High-throughput screening (HTS) is a popular approach to assessing ENM cytotoxicity that offers the opportunity to rapidly test in parallel a wide range of ENMs at multiple concentrations. However, current HTS approaches generally test one cell type at a time, which limits their ability to predict responses of complex microbial communities. In this study toxicity screening via a HTS platform was used in combination with next generation sequencing (NGS) to assess responses of bacterial communities from two aquatic habitats, Lake Michigan (LM) and the Chicago River (CR), to short-term exposure in their native waters to several commercial TiO_2_ nanomaterials under simulated solar irradiation. Results demonstrate that bacterial communities from LM and CR differed in their sensitivity to nano-TiO_2_, with the community from CR being more resistant. NGS analysis revealed that the composition of the bacterial communities from LM and CR were significantly altered by exposure to nano-TiO_2_, including decreases in overall bacterial diversity, decreases in the relative abundance of Actinomycetales, Sphingobacteriales, *Limnohabitans*, and *Flavobacterium*, and a significant increase in *Limnobacter*. These results suggest that the release of nano-TiO_2_ to the environment has the potential to alter the composition of aquatic bacterial communities, which could have implications for the stability and function of aquatic ecosystems. The novel combination of HTS and NGS described in this study represents a major advance over current methods for assessing ENM ecotoxicity because the relative toxicities of multiple ENMs to thousands of naturally occurring bacterial species can be assessed simultaneously under environmentally relevant conditions.

## Introduction

Engineered nanomaterials (ENMs) have at least one dimension less than 100 nm, which leads to unique chemical and physical properties, including high reactivity and conductivity. ENMs have a wide range of commercial applications, from pharmaceuticals and cosmetics to tools and electronics [1], and the nanotechnology industry has grown rapidly. For example, the manufacture of titanium dioxide (TiO_2_) nanoparticles is a significant niche industry and demand for these materials is strong. TiO_2_ nanoparticle (nano-TiO_2_) production was 50,400 tons in 2010, representing 0.7% of the overall TiO_2_ market. By 2015 production of nano-TiO_2_ is projected to increase to 201,500 tons [2]. Release of ENMs into the environment will be an inevitable consequence of high production and use [3], with models predicting the entry of ENMs into aquatic ecosystems [4]. There is growing concern that ENMs will have adverse effects on aquatic ecosystems, as previous studies have demonstrated ENM toxicity to aquatic organisms including fish, invertebrates and microorganisms (for reviews see [5], [6]). The toxicity of nano-TiO_2_, for example, is caused primarily by its photo-induced production of reactive oxygen species (ROS), which are powerful oxidizing agents that can damage a variety of cell components [7], [8].

One of the challenges to assessing ecological risks of ENMs is the incredible diversity of ENMs currently available and the rapid pace at which new ENMs are being developed [9], [10]. A recent survey identified 1,317 nanotechnology-based consumer products produced by 587 companies in 30 countries [11]. Assessing the relative toxicities of these materials will be challenging but is essential to development of ENMs with lower environmental impacts [12]. High-throughput screening (HTS) based on robotic liquid handling and fluorescence or absorbance assays has been recommended as a useful tool to assess ENM cytotoxicity [12]–[14]. HTS can be used to rapidly test a wide range of ENMs at multiple concentrations in parallel, and HTS studies have provided valuable information on the toxicity of a variety of ENMs [15]–[18]. However, current HTS approaches for assessing ENM toxicity generally test one cell type at a time, with bacteria commonly used as model organisms [15], [16], [18]–[21]. While single-species studies can assess comparative toxicities of different ENMs, these studies are limited in their ability to predict responses of multi-species bacterial communities. We recently used HTS to demonstrate that four bacterial species common in aquatic habitats varied dramatically in their responses to short-term exposure to nano-TiO_2_ [22], suggesting that bacterial community responses to ENM exposure are complex. Therefore, the goal of the present study is to use HTS to assess acute responses of aquatic bacterial communities to ENM exposure, using nano-TiO_2_ as a model ENM. Native bacterial communities were collected from two aquatic habitats that differed in physicochemical characteristics and bacterial community composition, Lake Michigan and the Chicago River. Bacterial communities were exposed in their native waters to four different commercial nano-TiO_2_ materials in a HTS format and bacterial viability was assessed using a standard fluorescence assay. In addition, the effects of nano-TiO_2_ on bacterial community composition were investigated using next generation sequencing of bacterial 16S rRNA genes.

## Materials and Methods

### Nano-TiO_2_ materials

Four nano-TiO_2_ materials were used: Aeroxide P25 (Evonik, Germany), Pigment White 6 (PW6, J.T. Baker, 4162–01), anatase nanopowder (ANP, Sigma, 637254), and rutile nanopowder (RNP, Sigma, 637262). We previously reported detailed characterizations of these materials [15]. Stock nano-TiO_2_ solutions were prepared at 2 g L^−1^ in milli-Q water (Millipore, 18 MΩ· cm) and sonicated in an ultrasonic bath at 42 kHz for 30 min. Stock solutions were further diluted in milli-Q water to the concentrations required for the toxicity assays.

### Sample collection

Water samples were collected from Lake Michigan (LM) and the Chicago River (CR). LM water was collected in Chicago, IL (42.01235°N/87.66092°W) on September 27, 2012. No permit is required for collecting water from LM. CR water was collected in Morton Grove, IL (42.05904°N/087.77234°W) on October 4, 2012. A permit for collecting water from CR was issued by the Forest Preserve District of Cook County. Water was collected from both locations in sterile Pyrex bottles, placed on ice for transport to the laboratory and analyzed that same day. Water samples were filtered using Grade 413 filter paper (VWR 28310–128). Characterization of these waters was reported previously [22].

### Toxicity screening via high throughput screening platform

For the HTS procedure bacterial viability was assessed using the Live/Dead BacLight kit (Molecular Probes). This assay allows determination of the relative abundance of viable bacterial cells based on the relative signal intensity of two fluorescent dyes: SYTO9, which is membrane permeable, and propidium iodide (PI), which is not membrane permeable and quenches SYTO9. A decrease in the ratio of fluorescent signals produced by SYTO9 (green) and PI (red) indicates a decrease in the number of viable bacterial cells, with viable cells defined as those with an intact cell membrane. The BacLight kit has been used previously in HTS assays by our group and others [15], [16], [18], [20].

In a preliminary experiment LM and CR water were added directly to microwell plates and bacterial viability was assessed with the BacLight kit. For both waters the SYTO9 signal was not above background (the signal produced by 0.2 µm filtered water) due to the low concentration of bacterial cells in these natural waters (approximately 200 cells ml^−1^ based on plate counts on nutrient agar). Therefore, bacterial communities in LM and CR water were concentrated by a factor of 200 via centrifugation at 3,452×g for 20 min followed by resuspension of cells in a smaller volume of the original source water, resulting in approximately 10^4^ cells ml^−1^ (based on plate counts on nutrient agar) and a SYTO9 signal that was 4 to 6 times higher than the background. This signal to background ratio provided an adequate dynamic range for assessing toxicity.

The HTS procedure described previously [15] was used with some modifications. Briefly, 25 µL of concentrated LM or CR bacterial communities and 25 µL nano-TiO_2_ suspensions (final concentrations of 0.5, 2.5, 5, 10, 25, 50 and 100 mg L^−1^) were added to wells of 384-well clear-bottom microplates using a robotic liquid handler (Biomek FX, Beckman Coulter) with 4 replicate wells per treatment. Plates were illuminated with a xenon arc lamp (Model 6271, Newport) and incubated at room temperature (approximately 22°C) with shaking at 300 rpm on an orbital shaker. To avoid evaporation each plate was covered with an ultraclear film (Axygen, UC-500) that had a negligible effect on light intensity and spectrum distribution [15]. Nano-TiO_2_ was also tested without illumination by covering a section of each plate with aluminum foil. After 1 hour incubation, 25 µL BacLight solution was robotically added to each well and plates were incubated for 15 min in the dark. Microplate reader (Synergy 4, BioTek) measured green (excitation 485 nm and emission 530 nm) and red (excitation 485 nm and emission 630 nm) fluorescence in each well. The relative abundance of viable bacterial cells within each well was expressed as a ratio of green to red fluorescence signals.

### Analysis of bacterial community composition

Next generation sequencing (NGS) of 16S rRNA genes was used to profile bacterial communities within selected wells immediately following toxicity assays. Specifically, four replicate wells of no treatment controls (which were not run through the HTS assay), and the illuminated 0 mg L^−1^ nano-TiO_2_ controls, 100 mg L^−1^ PW6 treatments and 100 mg L^−1^ P25 treatments for both LM and CR were analyzed (total of 32 samples). All material (75 µl) from each of these wells was collected and DNA was extracted with the WaterMaster DNA purification kit (Epicentre, Madison, WI). Suitability of extracted DNA for amplification was confirmed by PCR using bacterial primers 11F [23] and 519R [24] followed by gel electrophoresis.

DNA from each sample was sent to Research and Testing Laboratory (Lubbock, TX) for tag pyrosequencing. PCR amplification was performed using primers 28F (5′-GAGTTTGATCNTGGCTCAG-3′) [25] and 519R (5′-ACCGCGGCTGCTGGCAC-3′) [24]. Sequencing reactions utilized Roche 454 FLX (Roche, Indianapolis, IN) with Titanium reagents. Sequences were processed using MOTHUR (v.1.20.8) [26]. Barcodes and primers were removed and low-quality sequences (those with an average quality score <30, any ambiguous characters or homopolymers >8 bases) were removed. Remaining high quality sequences were aligned using the SILVA-compatible alignment database available within MOTHUR. Aligned sequences were trimmed to a uniform length of 250 base pairs and chimeric sequences were removed using UCHIME [27]. Sequences were classified by comparison to the MOTHUR-formatted version of the RDP training set (v.9) and any unknown (i.e., not identified as bacterial), chloroplast, mitochondrial, archaeal and eukaryotic sequences were removed. After pretreatment steps the LM data set included a total of 31,812 sequences for an average of 1,988 sequences per sample (+/−460) and the CR data set included a total of 31,253 sequences for an average of 1,953 sequences per sample (+/−253). Sequences were clustered into operational taxonomic units (OTUs) based on 97% sequence identity using the average neighbor algorithm. For comparisons of microbial community composition the number of sequences within each OTU was used as an indicator of the abundance of that OTU within a sample. As with all PCR-based methods, this approach is subject to potential biases inherent in PCR amplification. The OTU abundance data were imported into Primer V.6 (Primer-E Ltd., Plymouth, United Kingdom) and the data for each sample were normalized based on the total number of sequences obtained for that sample. A similarity matrix was created based on the Bray-Curtis index, which assigns every pair of samples a score between 0 and 1, where 0 represents no OTUs in common and 1 indicates two communities containing the same OTUs at the same abundance [28]. An ordination of the samples based on the Bray-Curtis similarity matrix was produced via nonmetric multidimensional scaling (nMDS) [29] run within Primer. The analysis of similarity (ANOSIM) routine in Primer was used to determine if there were statistically significant differences in composition between the experimental groups [30]. Diversity of bacterial communities was quantified based on Shannon index [31].

### Statistical Analyses

One-way ANOVA was used to test the effects of nano-TiO_2_ treatments on the relative abundances of viable cells, bacterial diversity, and relative abundances of specific bacterial phyla. Relative abundance data were arcsine square root transformed prior to ANOVA. In cases of significant treatment effects (p<0.05) Tukey's post hoc test was used for pairwise comparisons. ANOVAs and Tukey's tests were performed with Systat version 13 (Systat Software, Inc.).

### Data sharing

All of the sequence data analyzed in this paper can be downloaded from the National Center for Biotechnology Information (NCBI) Sequence Read Archive (SRA) with accession number SRP038125.

## Results

### Toxicity of nano-TiO_2_ Materials

**Figure 1:**
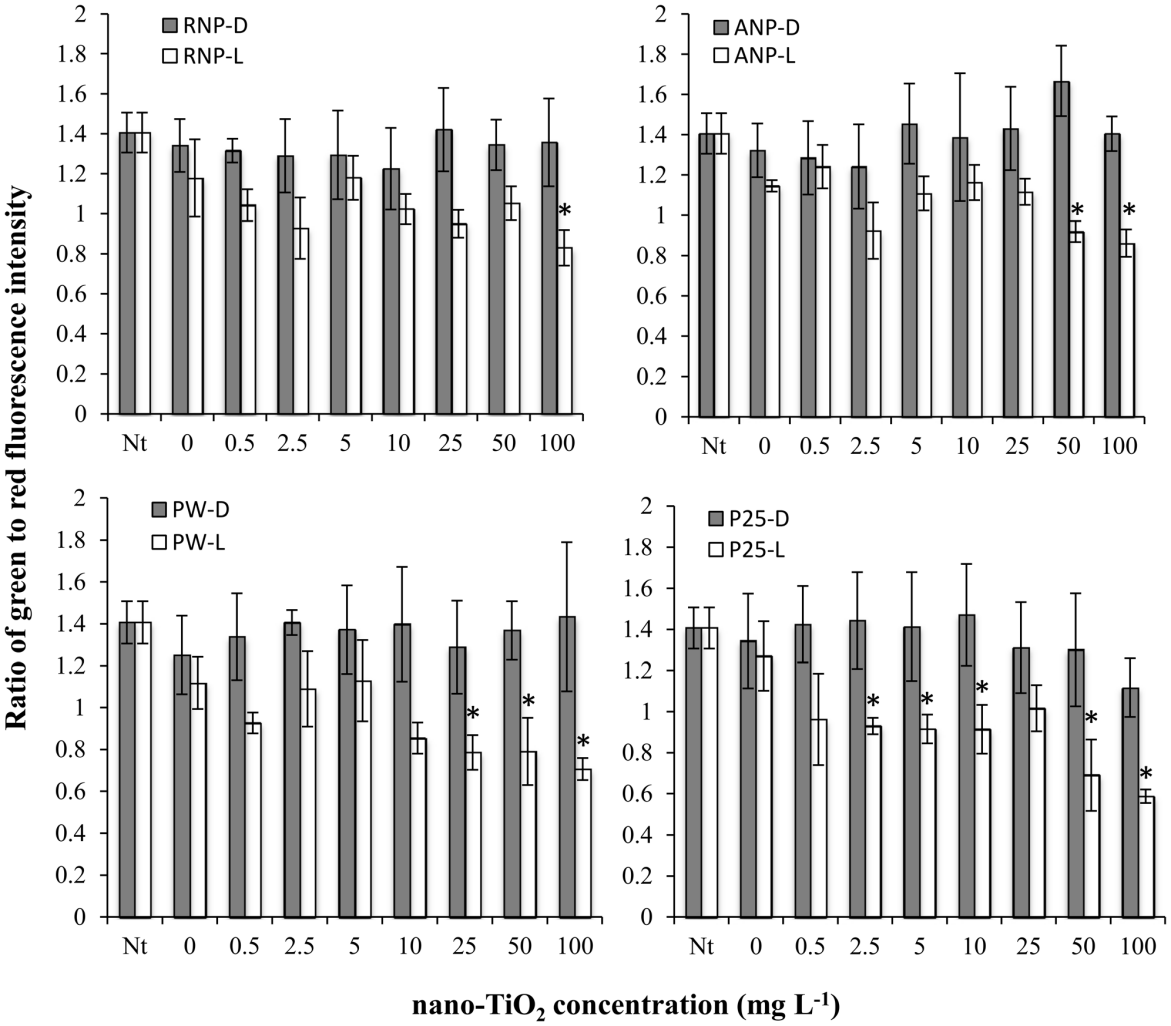
Ratio of live/dead bacteria in Lake Michigan water after 1 hour exposure in the dark (D) or under simulated sunlight (L) to one of four nano-TiO_2_ materials: rutile nanopowder (RNP), anatase nanopowder (ANP), Pigment White 6 (PW) or P25. Values are reported as mean of 4 replicates ± standard deviation. Nt: no treatment control prior to exposure. Data points marked by asterisks are significantly different (p<0.05) from the 0 mg L^−1^ nano-TiO_2_ control.

The toxicity of nano-TiO_2_ to bacterial communities from two natural surface waters, LM and CR, was assessed by HTS. These two waters had some differences in physicochemical characteristics: LM water had a dissolved organic carbon (DOC) concentration of 1.56 mg L^−1^, ionic strength of 4.75 mM and pH of 7.35, and CR water had a DOC concentration of 3.17 mg L^−1^, ionic strength of 12.16 mM and a pH of 7.63 [22]. Short term (1 h) exposure to several forms of nano-TiO_2_ with simulated solar illumination resulted in significant decreases in the relative abundance of viable bacterial cells within both LM and CR as indicated by decreases in green to red fluorescence ratios in the BacLight assay. For example, exposure of LM bacteria to RNP, ANP, PW6 and P25 with illumination had significant effects on the relative abundance of viable cells at concentrations of 100, 50, 25 and 2.5 mg L^−1^ respectively (Figure 1). P25 showed the highest toxicity to LM bacteria, with concentrations at or above 2.5 mg L^−1^ resulting in significant decreases in bacterial viability as compared to the 0 mg L^−1^ control. The order of toxicity for LM was P25>PW6>ANP>RNP. Without illumination none of the nano-TiO_2_ materials had significant effects on LM bacterial viability (Figure 1).

**Figure 2:**
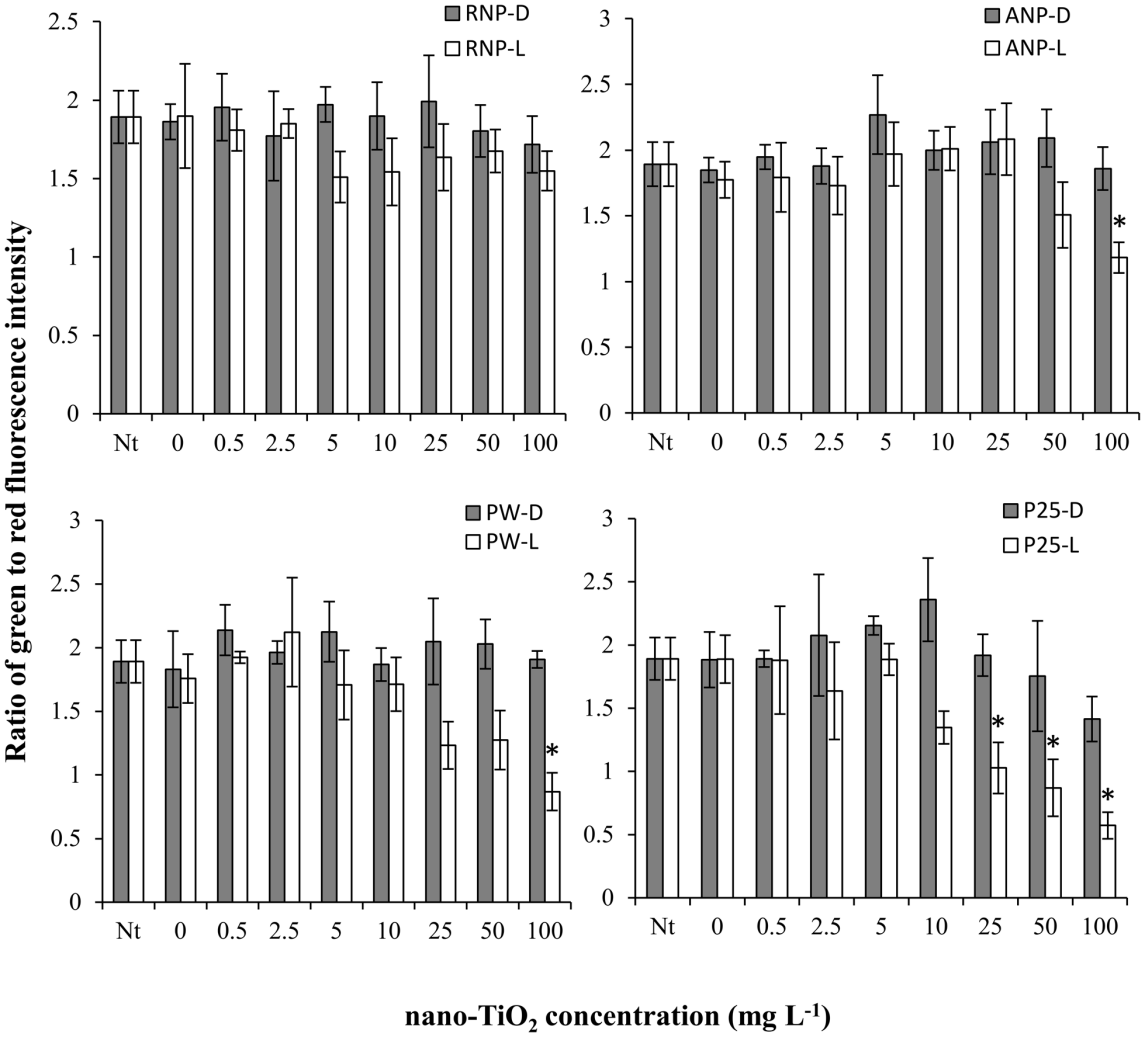
Ratio of live/dead bacteria in Chicago River water after 1 hour exposure in the dark (D) or under simulated sunlight (L) to one of four nano-TiO_2_ materials: rutile nanopowder (RNP), anatase nanopowder (ANP), Pigment White 6 (PW) or P25. Values are reported as mean of 4 replicates ± standard deviation. Nt: no treatment control prior to exposure. Data points marked by asterisks are significantly different (p<0.05) from the 0 mg L^−1^ nano-TiO_2_ control.

CR bacteria were less sensitive to nano-TiO_2_ than LM bacteria (Figure 2). For example, RNP with illumination had no significant effect on CR bacterial viability. ANP, PW6 and P25 with illumination did have significant effects on CR bacterial viability, although for ANP and PW6 only the highest concentration (100 mg L^−1^) resulted in significantly lower viabilities than the 0 mg L^−1^ control, while P25 concentrations at or above 25 mg L^−1^ resulted in significant decreases in viability. The order of toxicity for CR was P25>PW6 = ANP>RNP. None of the nano-TiO_2_ materials tested had significant effects on CR bacterial viability in the absence of illumination.

### Effects of nano-TiO_2_ on Bacterial Community Composition

**Figure 3:**
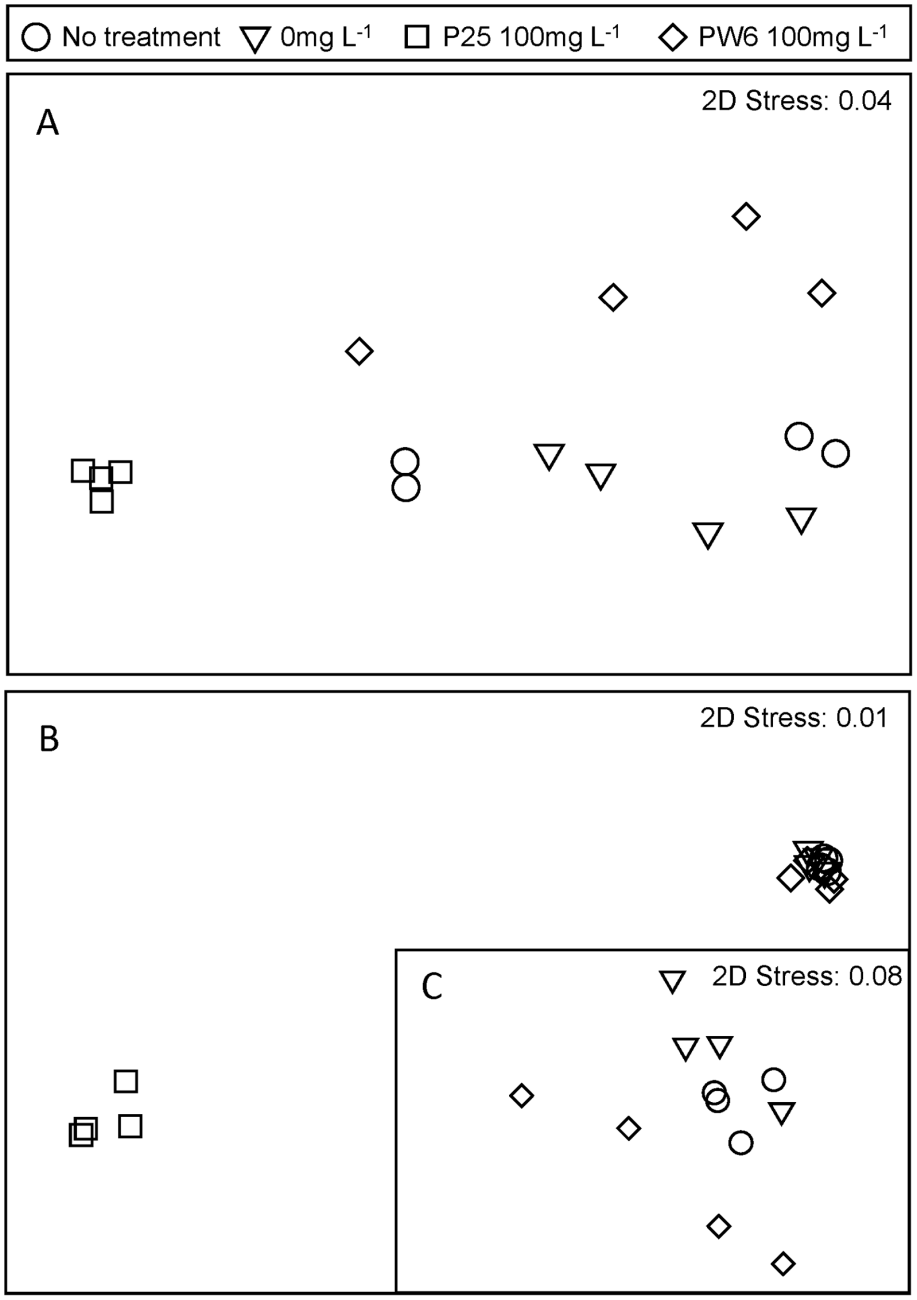
nMDS analysis of composition of bacterial communities from Lake Michigan water (A) and Chicago River water (B) prior to treatment (no treatment) and after 1 hour exposure to 0 mg L^−1^ nano TiO_2_, 100 mg L^−1^ Pigment White 6 (PW6) or 100 mg L^−1^ P25 under simulated sunlight. (C) nMDS for Chicago River water with 100 mg L^−1^ P25 treatment excluded. Bacterial community composition was analyzed based on tag pyrosequencing of 16S rRNA genes.

**Figure 4:**
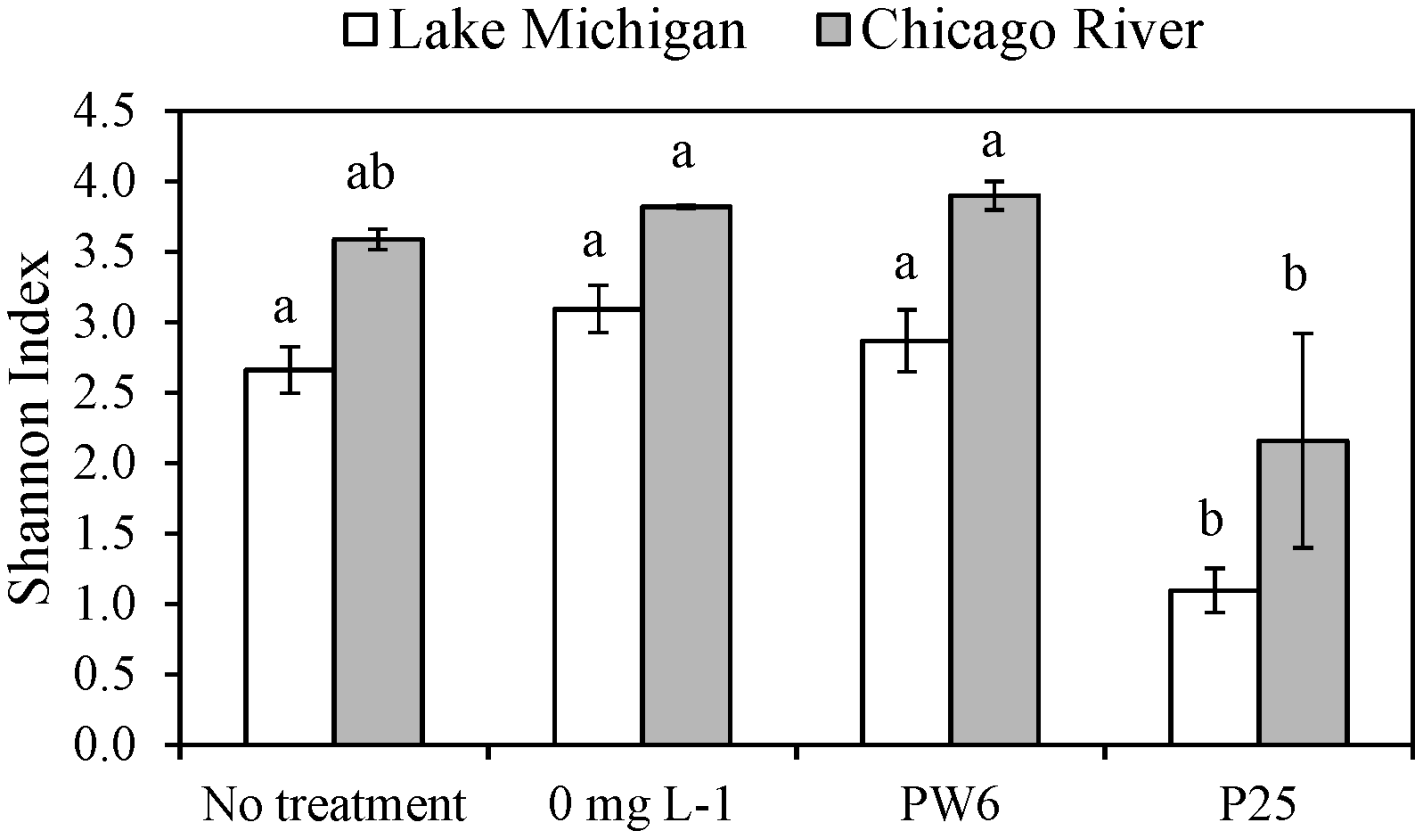
Diversity of bacterial communities from Lake Michigan and Chicago River prior to treatment (no treatment) and after 1 hour exposure to 0 mg L^−1^ nano TiO_2_, 100 mg L^−1^ Pigment White 6 (PW6) or 100 mg L^−1^ P25 under simulated sunlight . Bacterial community composition was analyzed based on tag pyrosequencing of 16S rRNA genes. ANOVA indicated significant treatment effects for Lake Michigan (p<0.001) and Chicago River (p<0.05) bacterial communities. Data points marked by different letters indicate significant differences between nano-TiO_2_ treatments within Lake Michigan or Chicago River communities based on Tukey's post hoc test.

Exposure of the LM bacterial community to 100 mg L^−1^ P25 resulted in a statistically significant shift in community composition as indicated by the separation of the P25 and 0 mg L^−1^ control samples in the nMDS ordination (Figure 3A) and the significant difference between these samples indicated by ANOSIM (Table 1). The difference in community composition between the P25 treatment and the 0 mg L^−1^ control was quite pronounced, as the average Bray-Curtis similarity between these treatments was only 0.14. P25 also resulted in a significant decrease in the diversity of the LM bacterial community, as indicated by the Shannon index (Figure 4). Exposure of the LM bacterial community to 100 mg L^−1^ PW6 also resulted in a statistically significant shift in composition as indicated by the separation of the PW6 and 0 mg L^−1^ control samples in the nMDS ordination (Figure 3A) and the significant difference between these samples indicated by ANOSIM (Table 1). However, the effect of PW6 was weaker than the effect of P25, with the PW6 treatment and the 0 mg L^−1^ control having an average Bray-Curtis similarity of 0.38. PW6 also did not have a significant effect on the diversity of the LM bacterial community (Figure 4). These results indicate that P25 exerted a stronger effect on the LM bacterial community than PW6, which is consistent with the viability results (Figure 1). Finally, the HTS procedure itself, i.e. exposure to simulated solar illumination for 1 h in microwell plates, did not have a significant effect on the composition of the LM bacterial community as indicated by the lack of separation between the 0 mg L^−1^ and no treatment controls in the nMDS ordination (Figure 3A), the lack of significant difference between these samples indicated by ANOSIM (Table 1), and the lack of significant difference in diversity between these samples (Figure 4). These results support the conclusion that the LM bacterial community changes observed in the P25 and PW6 treatments were caused by exposure to these materials and not by the conditions of the HTS assay.

**Table 1:** ANOSIM for bacterial community composition.

	Lake Michigan		Chicago River	
Comparison	R 1	p 2	R	p
No treatment, 0 mg L^−1^	0.063	0.371	0.167	0.114
P25 100 mg L^−1^, No treatment	1.000	0.029	1.000	0.029
P25 100 mg L^−1^, 0 mg L^−1^	1.000	0.029	1.000	0.029
PW6 100 mg L^−1^, No treatment	0.083	0.286	0.365	0.029
PW6 100 mg L^−1^, 0 mg L^−1^	0.292	0.029	0.521	0.029
P25 100 mg L^−1^, PW6 100 mg L^−1^	1.000	0.029	1.000	0.029

1 R statistic as defined by (29). R = 0 when there are no differences between groups. R approaches 1 as groups become more distinct. R = 1 when all samples within each group are more similar to each other than to samples in the other group.

2 Statistical significance of R statistic.

**Figure 5:**
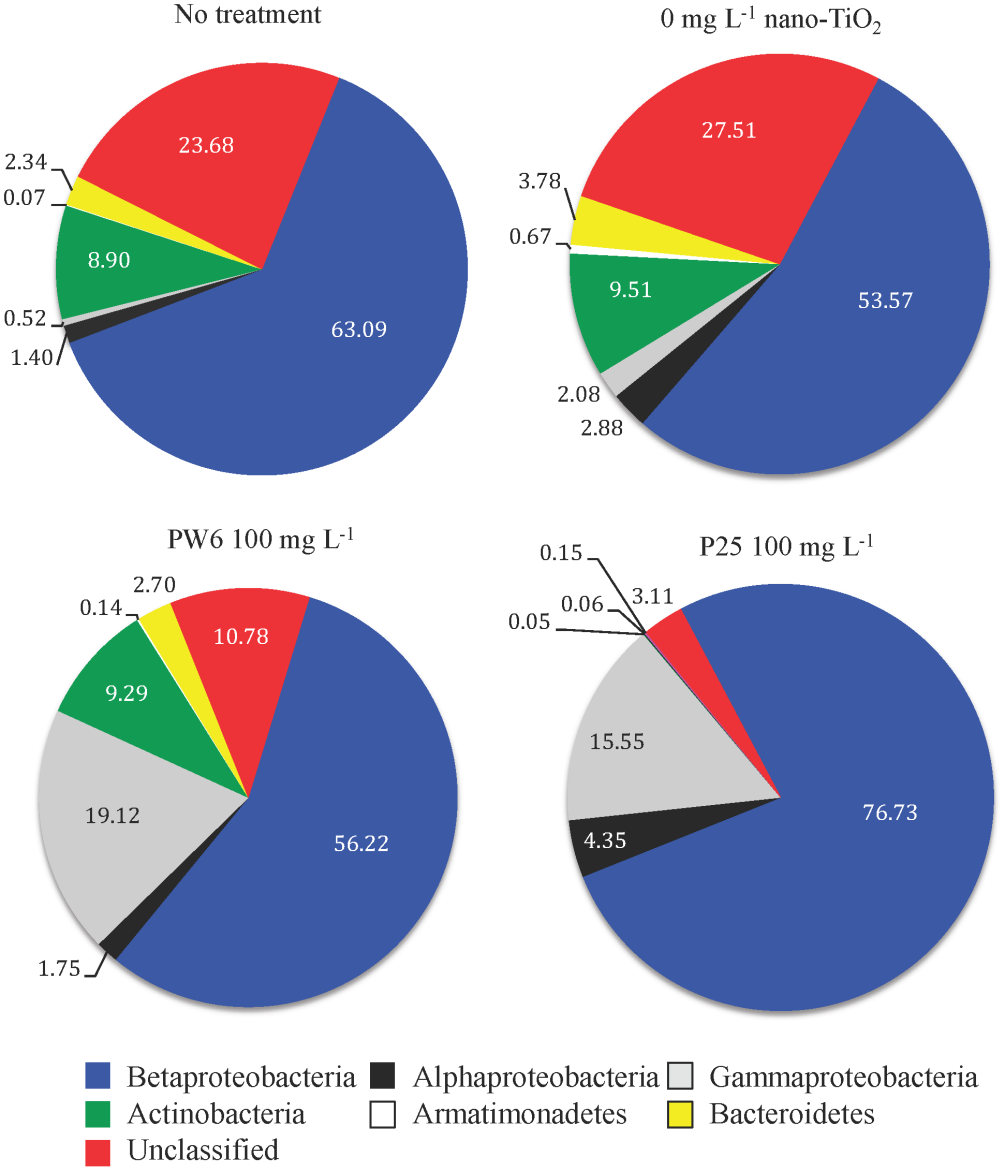
Mean relative abundances (in %; n = 4) of numerically dominant bacterial phyla and classes in Lake Michigan water prior to treatment (no treatment) and after 1 hour exposure to 0 mg L^−1^ nano-TiO_2_, 100 mg L^−1^ Pigment White 6 (PW6) or 100 mg L^−1^ P25 under simulated sunlight.

NGS also revealed some significant shifts in the relative abundance of sequences from several of the numerically dominant bacterial taxa within the LM bacterial community resulting from acute exposure to nano-TiO_2_ (Figure 5; Table 2). P25 exposure resulted in significant decreases in sequences from the phyla Actinobacteria and Bacteroidetes as well as a significant decrease in sequences from an unclassified bacterial phylum, with the Actinobacteria and Bacteroidetes sequences being almost completely eliminated from the LM community. Within the phylum Actinobacteria 93% of the sequences were identified as belonging to the order Actinomycetales, and this order decreased significantly in the P25 treatment. Within the phylum Bacteroidetes 78% of the sequences were identified as belonging to the order Sphingobacteriales, which also decreased significantly in the P25 treatment. As the relative abundance of these groups decreased with P25 treatment, the relative abundance of sequences from the classes Betaproteobacteria and Gammaproteobacteria increased within the LM community, although due to high variability these increases were not statistically significant. Within the Betaproteobacteria 57% of the sequences were identified as belonging to the genus *Limnobacter* and 36% were from the genus *Limnohabitans*. *Limnobacter* did show a statistically significant increase in the P25 treatment, whereas *Limnohabitans* showed a significant decrease. Exposure of the LM bacterial community to PW6 also resulted in some significant changes in the composition of the community, including decreases in Armatimonadetes and the unclassified bacterial phylum, although both decreases were less pronounced than for P25 (Table 2).

**Table 2:** ANOVA results for relative abundance of sequences from predominant bacterial taxa from Lake Michigan.

	No treatment			0 mg L^−1^			PW6 100 mg L^−1^			P25 100 mg L^−1^			
Taxa	Mean^2^	SE^3^		Mean	SE		Mean	SE		Mean	SE		p^1^
Class Betaproteobacteria	63.09	7.53	a^4^	53.57	3.41	a	56.22	11.93	a	76.73	13.15	a	0.288
Genus *Limnobacter*	27.20	14.83	a	16.97	7.04	a	10.03	4.27	a	74.96	12.54	b	0.008
Genus *Limnohabitans*	31.72	6.67	a	30.65	3.29	a	37.64	11.29	a	1.18	0.57	b	0.001
Class Alphaproteobacteria	1.40	0.24	a	2.88	0.44	a	1.75	0.70	a	4.35	1.30	a	0.080
Class Gammaproteobacteria	0.52	0.19	a	2.08	1.96	a	19.12	17.86	a	15.55	15.12	a	0.602
Phylum Actinobacteria	8.90	2.47	a	9.51	0.92	a	9.29	2.68	a	0.05	0.05	b	0.000
Order Actinomycetales	8.56	2.30	a	9.45	0.95	a	9.04	2.59	a	0.05	0.05	b	0.000
Phylum Armatimonadetes	0.07	0.02	a	0.67	0.27	b	0.14	0.06	a	0.06	0.06	a	0.009
Phylum Bacteroidetes	2.34	0.45	a	3.78	1.47	a	2.70	0.81	a	0.15	0.09	b	0.006
Order Sphingobacteriales	1.65	0.28	a	2.90	1.10	a	2.26	0.80	a	0.14	0.09	b	0.010
Unclassified Bacterial Phylum	23.68	5.20	a	27.51	1.31	a	10.78	2.75	b	3.11	1.69	b	0.000

1 p value based on one-way ANOVA.

2 Mean value (n = 4).

3 Standard error (n = 4).

4 Data points in the same row with different letters are significantly different based on Tukey's post-hoc test.

The CR bacterial community also responded to P25 treatment with a statistically significant shift in community composition, as indicated by the separation of the P25 and 0 mg L^−1^ control samples in the nMDS ordination (Figure 3B), the significant difference between these samples indicated by ANOSIM (Table 1) and a significant decrease in diversity for the P25 samples as compared to the 0 mg L^−1^ control samples (Figure 4). The difference in composition between the P25 and 0 mg L^−1^ treatments was dramatic, with an average Bray-Curtis similarity of 0.12. The CR bacterial community also responded to PW6, with a significant difference in composition between the PW6 treatment and the 0 mg L^−1^ control (Table 1). However, the response of the CR bacterial community to PW6 was weaker than the response to P25, with an average Bray-Curtis similarity between PW6 and 0 mg L^−1^ treatments of 0.55 and no significant change in diversity (Figure 4). In fact the shift in CR bacterial community composition caused by PW6 exposure was not evident in the nMDS plot due to the much greater shift caused by P25 (Figure 3B); but when the CR data were re-plotted without the P25 samples, separation between the PW6 and 0 mg L^−1^ treatments could be distinguished (Figure 3C). The weaker response of the CR bacterial community to PW6 as compared to P25 is consistent with the viability results (Figure 2). Finally, the HTS procedure itself did not have a significant effect on the composition of the CR bacterial community as indicated by the lack of separation between the 0 mg L^−1^ and no treatment controls in the nMDS ordination (Figure 3B and C), the lack of significant difference between these samples indicated by ANOSIM (Table 1), and the lack of significant difference in diversity between these samples (Figure 4). This result again supports the conclusion that the changes in CR bacterial community composition observed in the P25 and PW6 treatments were caused by exposure to nano-TiO_2_.

**Figure 6:**
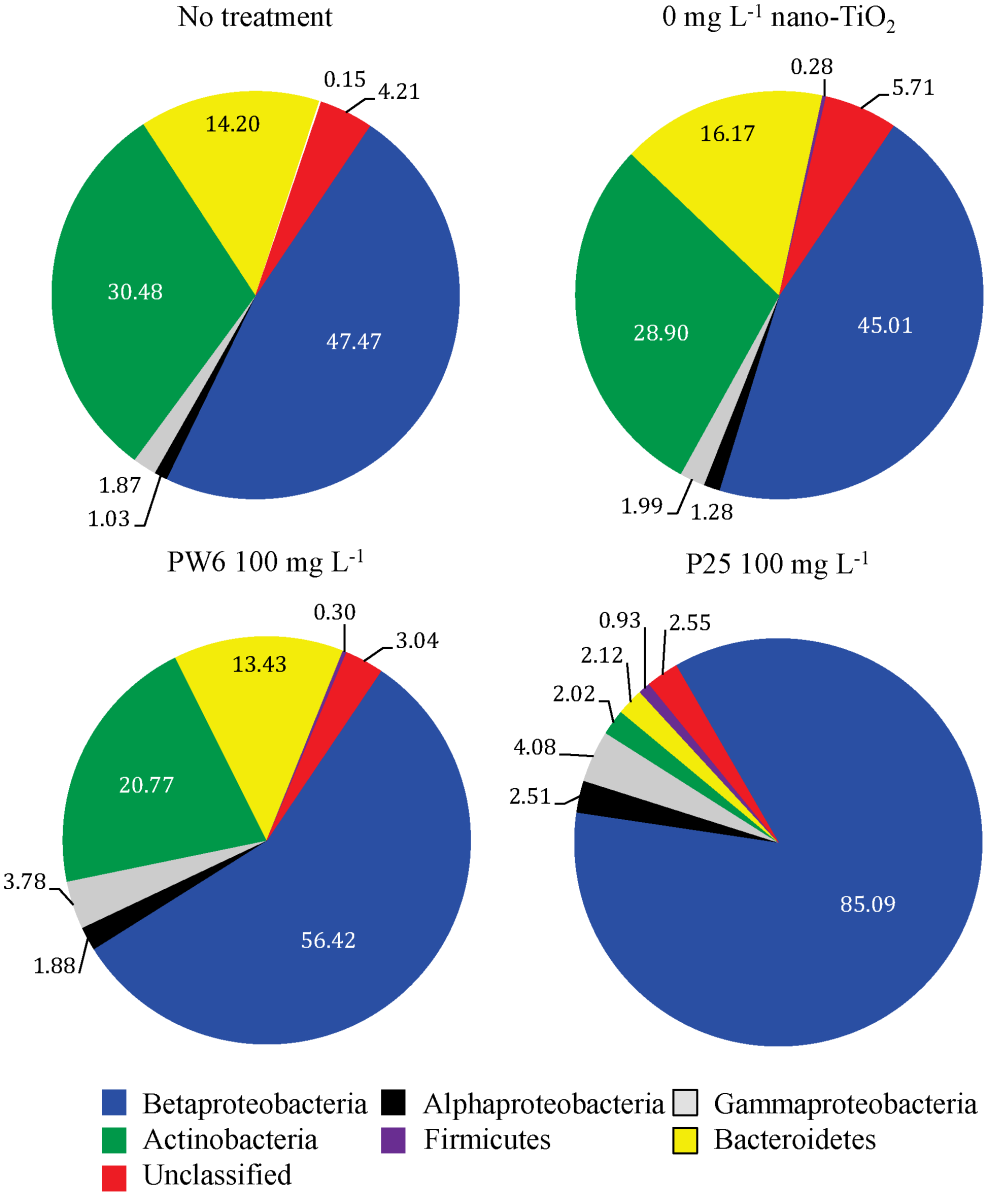
Mean relative abundances (in %; n = 4) of numerically dominant bacterial phyla and classes in Chicago River water prior to treatment (no treatment) and after 1 hour exposure to 0 mg L^−1^ nano-TiO_2_, 100 mg L^−1^ Pigment White 6 (PW6) or 100 mg L^−1^ P25 under simulated sunlight.

NGS data demonstrated that P25 exposure resulted in some significant taxonomic changes in the CR bacterial community as compared to the 0 mg L^−1^ control, including a significant increase in the relative abundance of sequences from the class Betaproteobacteria and significant decreases in the relative abundance of sequences from the phyla Actinobacteria and Bacteroidetes (Figure 6; Table 3). Within the Betaproteobacteria 31% of the sequences were identified as belonging to the genus *Limnobacter* and 46% were identified as belonging to the genus *Limnohabitans*, and the P25 treatments showed a significant increase in *Limnobacter* and a significant decrease in *Limnohabitans* as compared to the 0 mg L^−1^ control. Within the Actinobacteria 95% of the sequences were identified as belonging to the order Actinomycetales, which showed a significant decrease in the P25 treatment. Finally, within the Bacteroidetes 62% of the sequences were identified as belonging to the order Sphingobacteriales and 35% to the genus *Flavobacterium*, and both of these taxa decreased significantly in the P25 treatment. In contrast to P25, PW6 treatment did not result in dramatic changes in taxonomic composition of CR bacterial community, with the only significant changes being decreases in the order Actinomycetales and the genus *Flavobacterium*.

**Table 3:** ANOVA results for bacterial phyla from Chicago River.

	No treatment			0 mg L^−1^			PW6 100 mg L^−1^			P25 100 mg L^−1^			
Taxa	Mean^2^	SE^3^		Mean	SE		Mean	SE		Mean	SE		p^1^
Class Betaproteobacteria	47.47	0.45	a4	45.01	1.84	a	56.42	0.38	a	85.09	5.82	b	0.000
Genus *Limnobacter*	1.03	0.49	a	2.78	0.96	a	3.01	1.21	a	65.14	14.67	b	0.000
Genus *Limnohabitans*	35.70	0.71	a	30.61	0.49	a	35.90	1.60	a	6.40	2.81	b	0.000
Class Alphaproteobacteria	1.03	0.12	a	1.28	0.17	a	1.88	0.35	a	2.51	0.67	a	0.053
Class Gammaproteobacteria	1.87	0.08	a	1.99	0.44	a	3.78	0.99	a	4.08	1.89	a	0.482
Phylum Actinobacteria	30.48	0.85	a	28.90	1.83	a	20.77	0.73	b	2.02	0.84	c	0.000
Order Actinomycetales	29.00	0.77	a	27.71	1.74	a	19.74	0.82	b	1.97	0.81	c	0.000
Phylum Bacteroidetes	14.20	0.70	a	16.17	0.79	a	13.43	1.21	a	2.12	0.90	b	0.000
Order Sphingobacteriales	7.58	1.14	a	9.57	0.70	a	10.34	0.89	a	1.41	0.69	b	0.000
Genus *Flavobacterium*	6.37	1.19	a	6.21	0.27	a	2.81	0.48	b	0.46	0.21	c	0.000
Phylum Firmicutes	0.15	0.10	a	0.28	0.06	a	0.30	0.16	a	0.93	0.51	a	0.351
Unclassified Bacterial Phyla	4.21	0.34	a	5.71	0.64	a	3.04	0.49	a	2.55	1.13	a	0.056

1 p value based on one-way ANOVA.

2 Mean value (n = 4).

3 Standard error (n = 4).

4 Data points in the same row with different letters are significantly different based on Tukey's post-hoc test.

## Discussion

The results of this study indicate that short-term exposure of bacterial communities from two aquatic habitats, Lake Michigan (LM) and the Chicago River (CR), to several different forms of nano-TiO_2_ in their native waters under simulated solar illumination resulted in significant decreases in the relative abundance of viable bacterial cells. There were some significant differences and some interesting similarities in the responses of the bacterial communities from LM and CR to nano-TiO_2_ exposure. The communities from CR were generally less sensitive to nano-TiO_2_ than the communities from LM, with LM communities showing reduced viability in response to lower doses of both PW6 and P25 than the CR communities. This difference in sensitivity may have been due to the water matrix; we have demonstrated previously that when tested individually several bacterial species were more sensitive to nano-TiO_2_ in LM water than in CR water [22]. CR water has a higher ionic strength because of the higher level of dissolved salts (the concentration of chloride ions is 10 times greater in CR than in LM) and more importantly CR water has a higher concentration of DOM than LM water (the DOC of CR is twice that of LM), both of which can affect the behavior of nano-TiO_2_ materials. For example, higher ionic strength can lead to increased aggregation of nano-TiO_2_ [32], which may decrease production of reactive oxygen species (ROS). Our previous work has shown that DOM can also reduce the cytotoxicity of nano-TiO_2_ via three possible mechanisms: creating a physical barrier between bacteria and TiO_2_, absorbing UV, and ROS quenching [15]. These results suggest that the chemical composition of CR water may have contributed to lower nano-TiO_2_ toxicity. However, it is also possible that the differences between the CR and LM bacterial community responses to nano-TiO_2_ were related to differences in the properties of the communities themselves. For example, the LM community initially had a much higher relative abundance of an unclassified bacterial phylum, and this phylum proved to be especially sensitive to P25. In addition, previous exposure to pollutants may have played a role in CR and LM bacterial community responses to nano-TiO_2_. CR is a highly urbanized river with 30 combined sewer overflows (CSOs) that release urban runoff and untreated wastewater directly to the river during high rainfall events, while there are no CSO or wastewater effluent discharges to LM in the state of Illinois [33]. Concentrations of PCBs and chlorinated pesticides exceeding EPA standards have been detected in CR, with CSOs and urban runoff identified as likely sources, but these pollutants have not been detected in the water column at our LM sampling site [34]. The concentrations of nano-TiO_2_ and other ENMs at these sites are unknown, as these emerging pollutants are not currently monitored at these sites; but wastewater has been identified as a source of ENMs [35], [36], which suggests that ENM concentrations are likely to be higher within CR than LM due to the differences in wastewater inputs between these two sites. Therefore, the high concentration of anthropogenic pollutants in CR, possibly including nano-TiO_2_ and other ENMs, may have selected for a bacterial community that was dominated by more stress tolerant taxa, resulting in a community that was more resistant to nano-TiO_2_ exposure in our study. This hypothesis is supported by our previous work demonstrating that bacterial taxa can vary dramatically in their sensitivity to acute nano-TiO_2_ exposure, with some taxa showing high levels of resistance [22].

Despite the observed differences in overall sensitivity to nano-TiO_2_, when the LM and CR bacterial communities were exposed to a high concentration of the most toxic form of nano-TiO_2_ (P25) both showed significant shifts in the taxonomic composition of the communities and significant decreases in bacterial diversity as indicated by NGS analysis. It should be noted that the NGS assay is based on amplification of DNA by PCR, and it is possible to amplify DNA from cells that are no longer viable (e.g. that have a ruptured cell membrane) as long as the segment of DNA targeted by the PCR assay is still intact. Therefore, lack of detection of a cell by the NGS assay requires that the cell's DNA has been degraded to a point that it is not amplifiable by PCR. Previous studies have demonstrated the ability of nano-TiO_2_ to rapidly damage cellular DNA through the production of ROS [37]. Therefore, a decrease in the abundance of specific bacteria in our study implies not only a loss of cell viability but a degradation of cellular DNA.

The specific changes in bacterial community composition were remarkably similar for the LM and CR communities. Specifically, in both the LM and CR communities P25 exposure caused significant decreases in the relative abundances of Actinomycetales, Sphingobacteriales and *Limnohabitans*, and a significant increase in the relative abundance of *Limnobacter*. The CR community also showed a significant decrease in *Flavobacterium*, whereas *Flavobacterium* sequences were only occasionally detected within the LM community (less than 0.1% of the total sequences). Actinomycetales, Sphingobacteriales, *Flavobacterium* and *Limnohabitans* represent a phylogenetically diverse collection of bacteria, but they have all been shown by previous studies to be abundant and broadly distributed in freshwater ecosystems [38]–[41], so their predominance in the LM and CR communities agrees with the current understanding of the ecology of bacterioplankton communities. The bacterial taxa Actinomycetales, Sphingobacteriales, *Flavobacterium* and *Limnohabitans* all include heterotrophic organisms that utilize a variety of organic compounds. Bacteria from the genus *Limnohabitans* utilize algal-derived organic material as a substrate for growth [42]. Bacteria from the genus *Flavobacterium* are associated with the metabolism of carbohydrates and polysaccharides [43], including cellulose [44], [45]. Some aquatic Actinomycetes have been shown to produce cellulase and chitinase [46] and the *Sphingobacteriales* are known for their ability to metabolize complex organic compounds, including herbicides and antimicrobial compounds [47]. Therefore, the observed decreases in all of these taxa with acute exposure to nano-TiO_2_ suggest that nano-TiO_2_ may have the potential to disrupt carbon cycling processes in aquatic ecosystems.

Because the photo-toxicity of nano-TiO_2_ is based on the production of highly reactive ROS that can damage multiple cell structures [7], [8], it is not surprising that nano-TiO_2_ had a negative effect on a wide range of bacteria taxa. What is noteworthy is that *Limnobacter* was the only bacterial taxa identified in our study that showed a statistically significant increase in relative abundance with nano-TiO_2_ exposure, and that this increase was observed for both the CR and LM communities, even though they initially had very different abundances of this organism. *Limnobacter* started out as one of the dominant components of the LM community, accounting for 27% of the total sequences, and it increased to 75% of total sequences after one hour of exposure to nano-TiO_2_. In contrast *Limnobacter* was a very minor component of the initial CR community (1% of total sequences) whose relative abundance increased dramatically (65% of the total sequences) after one hour of exposure to nano-TiO_2._ NGS data provide insight into the abundance of bacterial taxa based on relative rather than absolute gene abundance, so it is not possible to determine from these data if the increases in relative abundance of *Limnobacter* represents increased growth of this organism in the presence of nano-TiO_2_ (i.e. the actual abundance of *Limnobacter* is increasing) or simply higher resistance to the toxic effects of nano-TiO_2_ (i.e. the relative abundance of *Limnobacter* is increasing because other more sensitive taxa are being killed). Given the short exposure time and the dramatic decreases in overall numbers of viable cells that we observed, higher resistance without additional growth seems like the most likely explanation for the increased relative abundance of *Limnobacter*. However, previous work by our group using the same HTS platform demonstrated that several bacterial species showed enhanced growth when exposed to nano-TiO_2_ in water from CR [22], so it is possible that the increase in relative abundance of *Limnobacter* could represent higher resistance coupled with enhanced growth. Whichever scenario is correct, our data demonstrate that *Limnobacter* was more resistant to the cytotoxic effects of nano-TiO_2_ than the other predominant bacterial groups in the LM and CR communities. The genus *Limnobacter* was first identified as a novel group of Betaproteobacteria in 2001 in a study that enriched for thiosulfate oxidizing bacteria from the sediment of a freshwater lake [48] and it has since been detected in the Baltic Sea and shown to degrade phenol [49]. Organisms within this genus are Gram-negative, aerobic heterotrophs that can grow chemolithoheterotrophically using thiosulfate as an energy source [48]. It is unclear at this stage why *Limnobacter* would be especially resistant to nano-TiO_2_, but this question warrants further study. The results of this study do demonstrate that bacterial taxa can vary significantly in their responses to nano-TiO_2_ exposure. This result is significant as it suggests that nano-TiO_2_ in the environment has the potential to act as a selective agent, decreasing the diversity and altering the composition of aquatic bacterial communities, which could have implications for the stability and function of aquatic ecosystems.

Our results also demonstrate that different types of nano-TiO_2_ vary in their toxicity to complex, multi-species aquatic bacterial communities. Specifically, the relative toxicities of the four tested nano-TiO_2_ materials were P25>PW6>ANP>RNP, which corresponds to the pattern we observed previously with *E. coli* [15] and to the relative photoactivity of these materials [15]. Since there was no effect of any of the nano-TiO_2_ materials on the CR or LM bacterial communities in the dark, our results also demonstrate that the toxicity of nano-TiO_2_ to aquatic bacterial communities is driven by its photoactivity, which concurs with findings from previous studies using single microbial species [15], [50], [51].

LM and CR bacteria showed significant decreases in viability at P25 concentrations of 2.5 and 25 mg L^−1^ respectively, which are higher than predicted concentrations for aquatic ecosystems [4]. However, these concentrations are relevant to possible environmental exposures. For example, Ti has been detected in the mg L^−1^ range in domestic wastewater [35], and there are multiple scenarios under which domestic wastewater is directly released to the environment. For example, in the United States untreated wastewater can be released to the environment via combined sewer overflows as well as sewer infrastructure failures or leaks. The U.S. EPA has predicted that the percentage of U.S. sewer pipes that will be in “poor,” “very poor,” or “life elapsed” conditions will increase from 23% in 2000 to 45% in 2020 [52], suggesting that sewer system failures and leaks are likely to increase in frequency in the U.S. In addition, the long-term release of low concentrations of nano-TiO_2_ to aquatic ecosystems will lead to accumulation of nano-TiO_2_ in sediment, and storms or other physical disturbance events could resuspend nano-TiO_2_ from sediments resulting in temporarily high concentrations of nano-TiO_2_ in the water column. Furthermore, the current exponential increase in nano-TiO_2_ production [53] suggests that nano-TiO_2_ concentrations in the environment are likely to surpass predicted values. Finally, it is important to note that this study assessed only the potential for nano-TiO_2_ to kill bacteria by rupturing their cell membrane. It is possible that lower concentrations of nano-TiO_2_ could trigger sub-lethal bacterial responses, such as changes in metabolic activity, gene expression, or bacterial defense systems, which could be relevant to the potential ecological effects of nano-TiO_2_ exposure. Further work is needed to assess these potential sub-lethal effects.

One limitation of the approach described in this study was the need to concentrate the cells from the natural waters by centrifugation prior to the HTS assay. Concentrating the cells was necessary to achieve a cell density adequate to produce a fluorescent signal with the BacLight kit that was significantly above the background. Specifically, cells were concentrated to 10^4^ cells ml^−1^. This cell density is several orders of magnitude lower than the cell densities generally used for HTS assays with pure cultures [15], [16], [18], [22], and it is not unrealistic for natural surface waters, as cell densities above 10^4^ cells ml^−1^ have been reported for a variety of surface waters based on plate count assays [54]–[56]. In cases where cell densities in natural waters are above this threshold, concentrating cells should not be necessary to run this assay. For the surface waters sampled in this study, concentrating cells was necessary, and may have also resulted in an increased concentration of some insoluble materials, such as organic particulates. These insoluble materials could reduce the toxicity of nano-TiO_2_ by scavenging ROS or absorbing UV, suggesting that our results might be an underestimate of nano-TiO_2_ toxicity.

The results of this study demonstrate that high-throughput screening (HTS) analysis in combination with next generation sequencing (NGS) can be used to assess the responses of complex, multi-species aquatic bacterial communities to short term exposure to nano-TiO_2_ in their native waters. This novel approach represents a major advance over current HTS methods for assessing ENM ecotoxicity because the relative sensitivities of hundreds to thousands of naturally occurring bacterial species can be assessed simultaneously under environmentally relevant conditions. The HTS approach also offers some distinct advantages over larger scale mesocosm or field experiments because high-density microwell plates and liquid handling robots enable much more highly parallel experiments than would be possible with other approaches, permitting multiple ENMs to be tested at multiple concentrations with multiple microbial communities simultaneously.
